# Targeted next generation sequencing in Chinese colorectal cancer patients guided anti-EGFR treatment and facilitated precision cancer medicine

**DOI:** 10.18632/oncotarget.21349

**Published:** 2017-09-27

**Authors:** Helei Hou, Dong Liu, Chuantao Zhang, Yanxia Jiang, Guifang Lu, Na Zhou, Xiaonan Yang, Xiaoping Zhang, Zhuokun Li, Hongmei Zhu, Zhaoyang Qian, Xiaochun Zhang

**Affiliations:** ^1^ Department of Medical Oncology, The Affiliated Hospital of Qingdao University, Qingdao University, Qingdao, 266005, China; ^2^ Department of Pathology, The Affiliated Hospital of Qingdao University, Qingdao University, Qingdao, 266005, China; ^3^ Department of Gastroenterology, The First Affiliated Hospital of Xi'an Jiaotong University, Xi'an, 710061, China; ^4^ BGI-Qingdao Institute, Qingdao SINO-GERMAN Ecopark, Qingdao, 266555, China; ^5^ Department of Clinical Laboratory, BGI-Shenzhen, Shenzhen, 518083, China; ^6^ Binhai Genomics Institute, BGI-Tianjin, BGI-Shenzhen, Tianjin 300308, China

**Keywords:** next generation sequencing, colorectal cancer, genetic alteration, targeted therapy, personalized therapy

## Abstract

**Objective:**

Colorectal cancer (CRC) patients with both *RAS* and *BRAF* wild-type tumors determined by non-next generation sequencing (NGS) testing may still not respond due to the presence of additional mutated genes such as *PIK3CA* or *PTEN*. In this study, a broad, hybrid capture-based NGS assay was used to identify *RAS, BRAF* and additional targetable genetic alterations from Chinese CRC tissues.

**Methods:**

Fifty-seven cases of CRC were enrolled, and all the patients signed the informed consent. In total, 7708 exons of 508 tumor-related genes and 78 introns of 19 frequently rearranged genes were assessed for base substitutions, INDELs, copy number alterations, and gene fusions.

**Results:**

The study found that 50.9% (29/57) of the tumors harbored *KRAS* mutations, 3.5% (2/57) harbored *NRAS* mutations and 3.5% (2/57) harbored *BRAF* mutations. More specifically, 89.7% (26/29) of *RAS* mutations were located in codon 12. Except for *RAS* and *RAF*, anti-EGFR therapy response genetic mutations in *PTEN* (n=2) and *PIK3CA* (n=1) were found in 4.7% (3/64) of the samples. Actionable alterations were found in *HER2* (n = 7), *CCND2* (n = 2), *NF1* (n = 1), and *BRCA1* (n = 1).

**Conclusions:**

Our results illustrated that 82.5% (47/57) of the samples harbored at least one actionable genetic alteration identified by NGS. *HER2* amplifications or mutations, which were identified in 12.3% of the tissues, defined a unique molecular subtype of CRC. The study suggests that high-throughput NGS testing in CRC tissues is a comprehensive and efficient genomic profiling assay to guide personalized therapy.

## INTRODUCTION

Colorectal cancer (CRC) is one of the most common solid tumor cancer types worldwide. The discovery of mutant *KRAS* as a predictor of resistance to EGFR monoclonal antibodies resulted in major progression in the treatment of metastatic CRC [1]. However, less than 20% of CRC patients with KRAS wild-type tumors may have durable responses to anti-EGFR treatment [2]. Therefore, all patients with metastatic CRC should be genotyped for *KRAS, NRAS* and *BRAF* mutations [3, 4]. Patients with mutations in these genes may not benefit from anti-EGFR targeted therapy.

Studies have identified several genetic alterations, including *KRAS, NRAS* and *BRAF* mutations, which are associated with resistance to anti-EGFR agents. A retrospective analysis confirmed the negative effects of *KRAS, NRAS* and *BRAF* mutations on the outcome after cetuximab treatment. The research also found that *PIK3CA* exon 20 mutations were significantly associated with a low response rate [4]. Furthermore, loss of *PTEN* expression was associated with a negative outcome of cetuximab-based treatment, although this result needs to be validated in more prospective studies [5–7]. In 174 advanced CRC patients with synchronous or metachronous distant metastasis, *HER2* gene amplification was identified in 6.3% patients [8]. *HER2* amplifications induced the activation of downstream signaling and led to cetuximab or panitumumab resistance [9, 10]. To examine the effect of somatic genetic changes in CRC that caused a response to anti-EGFR antibody therapy, a study was conducted to complete exome sequencing and copy number analyses of patient-derived tumor grafts and targeted genomic analyses of tumors, all of which were *KRAS* wild-type [11]. This study detected alterations in all genes previously thought to be involved in EGFR therapeutic resistance: *NRAS* codon 12 or 61 mutations, *BRAF* V600E mutation, *MET* amplification, *HER2* amplification, *PIK3CA* exon 20 mutations and truncating or homozygous deletions of *PTEN*. Additionally, mutations in *HER2, EGFR, FGFR1, PDGFRA*, and *MAP2K1* were also identified as potential mechanisms of primary resistance to anti-EGFR therapy.

As the number of validated anti-EGFR efficiency related genomic alterations increased, multigene panel molecular assays are needed to guide CRC treatment [12]. Hybrid capture-based next-generation sequencing (NGS) enables the simultaneous detection of multiple mutations in multiple genes. More importantly, NGS could discover novel targeted alterations and new available agents that could potentially be used in cancer treatment [13]. Using formalin-fixed paraffin embedded (FFPE) tumor tissues, NGS was well applied to guide precision cancer medicine in diverse cancer types [14, 15]. Several studies have already evaluated the clinical application of NGS in CRC, using panels covering 20-183 genes [16, 17].

In this study, a 508 gene panel-based NGS assay was used to identify anti-EGFR sensitivity-related mutations along with additional targetable genetic alterations from 57 CRC tissues. Comprehensive molecular profiling by NGS assays facilitates personalized therapy for CRC patients, which might be widely applied in the future.

## RESULTS

### NGS assay and genetic alterations

**Figure 1:**
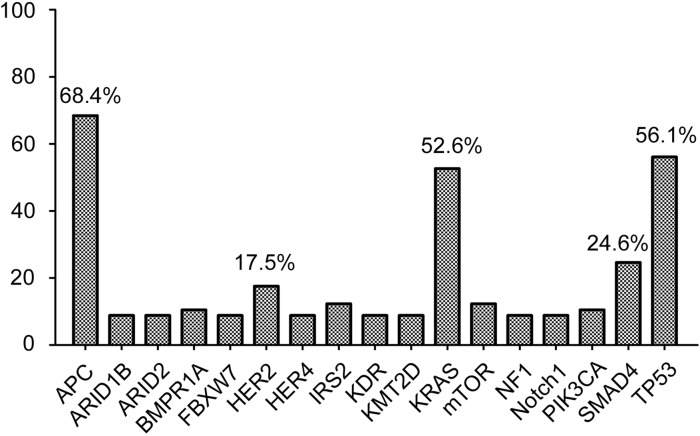
Cancer-related genes altered in more than 5 samples Seventeen mutant genes, including genetic alterations with or without available targeted agents, were observed in more than 5 samples. The top 5 most frequently mutant genes were *APC, TP53, KRAS, SMAD4* and *HER2*.

Fifty-seven CRC cases were selected for NGS assays, including 49 primary tumor tissue samples and 8 metastasis biopsies. The mean age was 60.4 years (range 35-83 years). Patient characteristics are listed in Supplementary Table 1. Approximately 14 days passed from DNA processing to the final report generation. The median average sequencing depth was 660.4±295.0 for tumor DNA from each sample. Genetic alterations were found in 204 genes in all 57 patients (mean 8.9±3.3, range 4-22). Seventeen genes, including those with targeted alterations and without available targeted agents, were observed in more than 5 samples (Figure 1). The most frequently mutated genes were *APC* (n=39, 68.4%), *TP53* (n=32, 56.1%), *KRAS* (n=30, 52.6%), SMAD4 (n=14, 24.6%) and *HER2* (n=10, 17.5%). Furthermore, 89.5% (51/57) of patients harbored at least one genetic alteration in *APC, TP53* or *KRAS*. The detailed sequencing data are provided in Supplementary Table 2.

### Molecular classification of CRC identified by NGS

**Figure 2:**
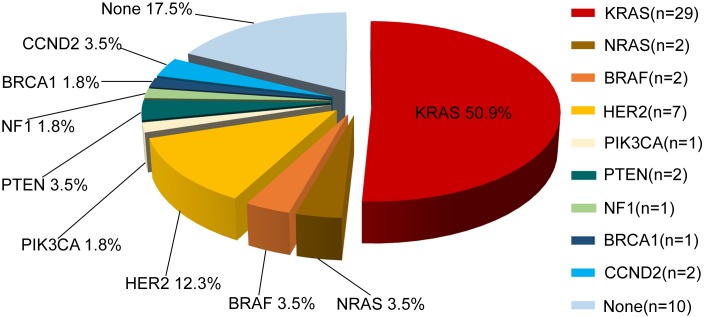
Molecular profiling of actionable genetic alterations in 57 CRC patients

In total, 47 of 57 (82.5%) patients had at least one potentially actionable alteration. The mean number of potentially actionable alterations per patient was 1.1 (1.1±0.8, range 0-4). Molecular classification of the 57 patients identified by the NGS assay is shown in Figure 2. The specific alterations that were observed in the most common actionable targets included the following: *KRAS* (n=29, 50.9%), *NRAS* (n=2, 3.5%), *BRAF* (n=2, 3.5%), *PIK3CA* (n=1, 1.8%), *PTEN* (n=2, 3.5%), *HER2* (n=7, 12.3%), NF1 (n=1, 1.8%), *BRCA1* (n=1, 1.8%) and *CCND2* (n=2, 3.5%) (Figure 2). Finally, 17.5% (10/57) of patients had no actionable somatic alterations.

**Figure 3:**
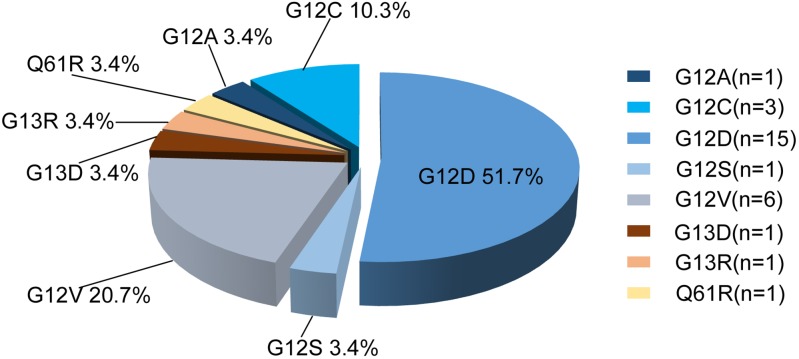
Distribution of *KRAS* mutation sites in 29 CRC specimens

The specific mutation sites of *KRAS* observed in 29 patients are shown in Figure 3. Of the *KRAS* mutations, 89.7% (26/29) were located in codon 12, 6.9% (2/29) were in codon 13 and 3.4% (1/29) were in codon 61.

### Clinical implications directed by the NGS assay

Considering the anti-EGFR therapy response and targeted drug options for the actionable alterations, clinical implications of the 57 CRC patients are shown in Table 1. Of the tumors harboring *KRAS, NRAS* or *BRAF* mutations, 57.9% (33/57) did not benefit from anti-EGFR agents, such as cetuximab or panitumumab. Furthermore, 17.5% (10/57) patients harboring somatic mutations or copy number variations in *PIK3CA*, *PTEN* (mutation or loss) and *HER2* (mutation or amplification) might be associated with a low response to anti-EGFR therapy. Finally, for the remaining 24.6% (14/57) of the patients, cetuximab or panitumumab could be used according to the NCCN guidelines.

**Table 1:** NGS results and clinical implication in 57 CRC patients

Actionable alterations in 57 patients	Response to anti-EGFR therapy
BRAF G469A + PI3KCA E545K	Resistant to anti-EGFR therapy: 33/57 (57.9%)
BRAF V600E	
KRAS G12A + PI3KCA E542K; KRAS G12C + PI3KCA E542K; KRAS G12V + PI3KCA E546K	
KRAS G12D + FBXW7 W237^*^	
KRAS G12V + mTOR S2215Y	
KRAS Q61R + PTEN loss+ HER2 T862A	
KRAS G12C/G12D/G12S/G12V/G13D/G13R	
NRAS G12D + NF1 F843Sfs^*^35 + ATM c.[3577-1G>T]	
NRAS Q61R	
PIK3CA H1047R	Associated with low-response to Anti-EGFR therapy: 10/57 (17.5%)
PTEN L100Tfs^*^	
PTEN loss + NF1 S1864Rfs^*^3 + NF1 D1866Rfs^*^17	
HER2 G776V + FBXW7 R658^*^	
HER2 V842I + PIK3CA E545K	
HER2 amplification	
HER2 amplification + ERG-TMPRSS2 fusion	
BRCA1 Q148^*^	Approved anti-EGFR therapy: 14/57 (24.6%)
CCND2 amplification	
NF1 R1306^*^ + EGFR amplification	
None actionable alterations detected	

### Association between actionable alterations

**Figure 4:**
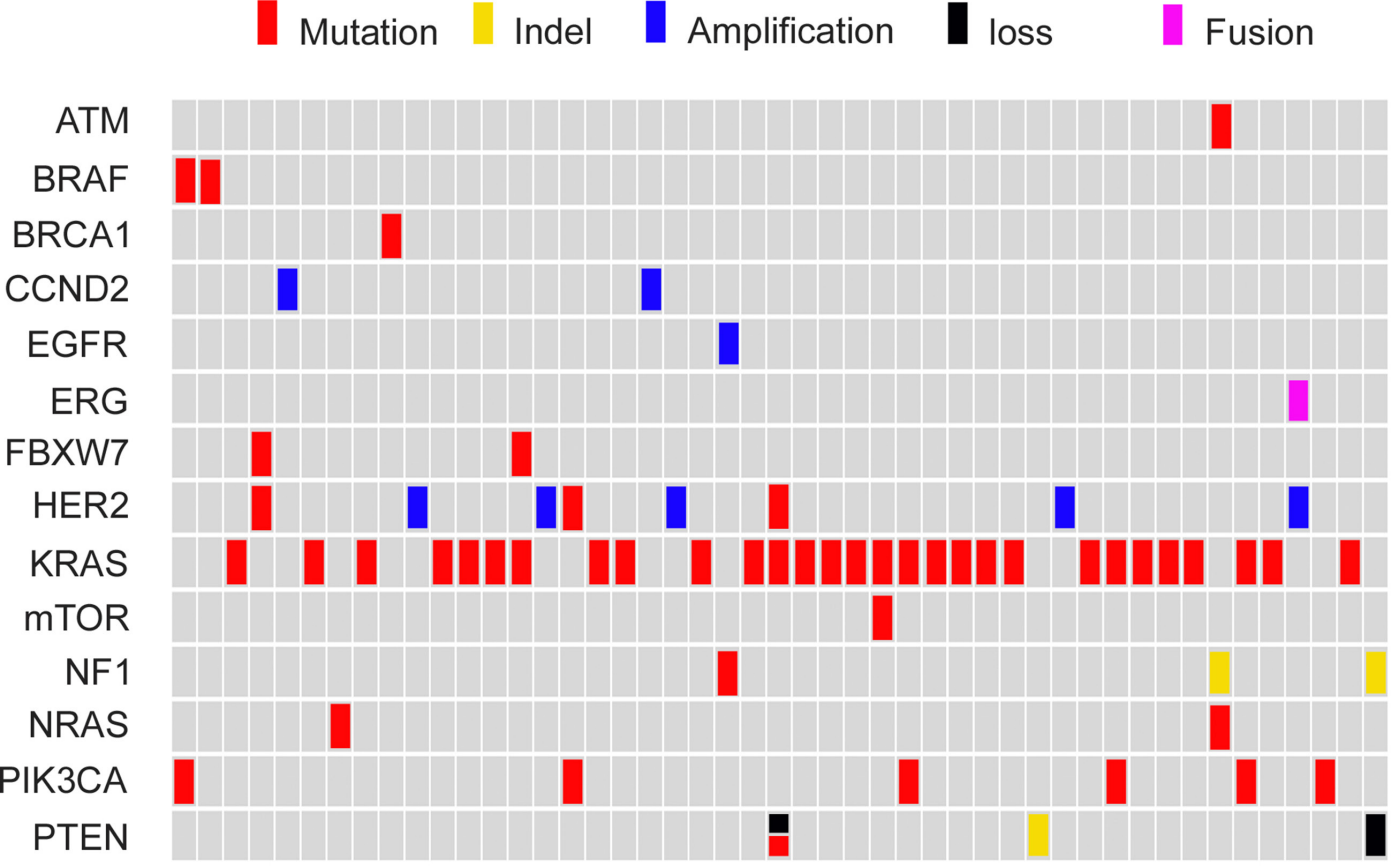
Comprehensive annotation of actionable genetic alterations identified by NGS assay in 57 CRC

Notably, more than one actionable alteration was identified in 22.8% (13/57) of the patients (Figure 4). Consequently, several targeted agents, including both approved drugs and experimental agents, might be simultaneously available for these patients. In this case, only the actionable alteration with the best clinical option, such as on-label agents will be exhibited in molecular classification (Figure 2). The additional overlapping actionable alterations included the following: *PIK3CA* (n=5), *FBXW7* (n=2), *NF1* (n=2), *PTEN* (n=1), *HER2* (n=1), *mTOR* (n=1), *ATM* (n=1), *EGFR* (n=1) and *ERG-TMPRSS2* (n=1) (Figure 4).

*KRAS* and *NRAS* mutations were mutually exclusive, as were *KRAS* and *BRAF* mutations, and *NRAS* and *BRAF* mutations (Figure 4). Of the KRAS mutant tumors, 20.7% (6/29) harbored *mTOR* pathway-related genetic alterations (*FBXW7, PTEN, PIK3CA*, and *mTOR*), of which *PIK3CA* exon 9 mutations accounted for half (n=3). One HER2 T862A mutation concurred with KRAS *Q61R*, while all cases of *HER2* amplification found in this research (8.8%, 5/57) were exclusive with mutations of *KRAS, NRAS* and *BRAF*.

## DISCUSSION

As more anti-EGFR resistance related and candidate therapeutically targeted genetic alterations are identified, comprehensive molecular diagnostic approaches are needed to match CRC patients with the appropriate therapies [13]. Here, we described a targeted sequencing test using broad, hybrid capture-based NGS technology across 508 cancer-related genes in Chinese CRC patients. The most frequently mutated genes identified in this study were *APC, TP53* and *KRAS*. In total, 89.5% of the patients harbored at least one genetic alteration in the above genes. Our results discovered that 82.5% of CRC patients harbored at least one actionable genetic alteration, which suggests that NGS could provide comprehensive and robust genetic alteration data to guide personalized CRC care. Several studies have already evaluated the clinical application of NGS in CRC [18–24]. Compared to these studies, the molecular profiling of representative actionable genetic mutations found in Chinese CRC patients was similar.

The major advantage of NGS is that multiple genetic alterations can be profiled within a single assay. Our research found that 57.9% of CRC harbor mutations in *KRAS, NRAS* or *BRAF*. Additionally, another 17.5% of patients with wild type versions of the above genes were identified with genetic mutations in *PTEN, PIK3CA* and *HER2*. Consequently, up to 75.4% of patients might not benefit from anti-EGFR therapy. Meta-analysis has suggested that mutations in *KRAS, NRAS, BRAF, PIK3CA* and non-functional *PTEN* predict resistance to anti-EGFR therapy [25]. Therefore, it is necessary to screen mutations in genes other than *RAS* and *BRAF* to optimize the identification of patients who will benefit from anti-EGFR treatment. Multiple gene panel analysis by NGS could be widely applied in the future to improve cancer therapy.

*HER2* amplifications or mutations are commonly found in a wide range of solid cancers. In this study, *HER2* amplifications and mutations were detected in 14.0% of the CRC patients. All of the *HER2* amplifications identified were exclusive with *KRAS, NRAS* and *BRAF*. In CRC, *HER2* amplifications induced the activation of downstream signaling even when cetuximab was bound to EGFR, thus leading to drug resistance [26]. *HER2* amplification was present in 6.0% to 13.8% of the tumors and was correlated with negative responses to anti-EGFR targeted therapy in *KRAS* wild-type CRC [10]. Therefore, *HER2* amplification contributes to the resistance of anti-EGFR therapy in partial *RAS* and *RAF* wild-type CRC patients and should be routinely tested. Pre-clinical and clinical data provide a rationale for the inclusion of trastuzumab, pertuzumab and pan-ERBB inhibitors in the treatment strategy for *HER2* amplification or mutation cancer patients. Response of CRC patients with *HER2* amplification or *HER2* overexpression to treatment with the combination of trastuzumab plus lapatinib was tested in a phase 2 clinical trial, the HERACLES trial [27]. Researchers screened 914 patients who were refractory to chemotherapy and had *KRAS* wild-type CRC, identifying 46 patients as having *HER2*-positive disease and enrolling 27 eligible patients into the study. Twenty patients (74.1%) achieved either a complete response, partial response, or stable disease. The median response duration was 9.5 months, median progression-free survival was 5.2 months, and median overall survival was 11.5 months. The phase II MyPathway trial evaluated agents targeting the *HER2, BRAF, Hedgehog*, or *EGFR* pathways in patients with advanced cancer. The most promising efficacy was seen among patients with *HER2*-amplified bladder, biliary, and CRC [28]. The HERACLES trial, as well as the MyPathway basket trials, showed the potential of *HER2* as a promising target in the treatment of CRC. *HER2* amplifications should be defined as a novel molecular subtype of advanced CRC.

*KRAS* and *NRAS* mutations are most commonly located in codons 12, 13 and 61, and lead to the constitutive activation of *RAS* [29]. Our analysis revealed that 57.9% of samples had mutations in the oncogenes *KRAS, NRAS* and *BRAF*, and the majority of *KRAS* mutations were found in codon 12. A previous study used the Ion PGM and AmpliSeq cancer panel to identify genetic mutations in 91 rectal cancer patients. Of the samples, 69.2% had mutations in the *RAS* signaling pathway [20]. Consistent with this study, concurrence of *KRAS, NRAS*, and *BRAF* mutations with PTEN/PI3K/AKT/mTOR pathway genetic alterations, including *PTEN, NF1, FBXW7, PIK3CA* and *mTOR*, were frequently detected in our research. No targeted therapies are available for *KRAS*-mutant CRC that progresses after all approved standard therapies have been given. Clinical trials showed that inhibitors of MEK1/2, downstream of the *RAS* pathway, had promising efficiency in *KRAS* mutant NSCLC and *NRAS* mutant melanoma [30, 31]. However, clinical activity with single MEK inhibitors had limited efficiency and acquired resistance was easily developed. *PI3K* signaling was important for cell survival in *NRAS* mutant melanoma when MEK was inhibited [32]. Combined targeting of the MEK/ERK and PI3K/mTOR pathways had antitumor activity and might serve as a therapeutic option in the treatment of *NRAS* mutant melanoma [32, 33]. Preclinical data supported dual targeted inhibition of MEK and one or more of the PI3K/AKT pathway effectors in metastatic CRC, which was superior to a single agent alone [34]. Future clinical trials with molecular stratification by NGS have the potential to improve outcomes for CRC patients, especially for patients with additional actionable alterations other than *RAS* and *RAF*.

In conclusion, our results showed that 82.5% of CRC tissues harbored at least one actionable genetic alteration identified by NGS testing. In addition to the 57.9% of CRC harboring *KRAS, NRAS* and *BRAF* mutations, another 17.5% patients with genetic mutations in genes including *PTEN, PIK3CA* and *HER2* might not benefit from anti-EGFR therapy. *HER2* amplification or mutation, which was identified in 12.3% of the tissues, defined a unique molecular subtype of CRC. This study highlights the potential of NGS to further help identify specific CRC patients who could benefit from anti-EGFR treatment. NGS provides a high-throughput and systematic method to identify all genetic alterations and to define novel molecular targets, which might be routinely used to guide precision cancer medicine in the future.

## MATERIALS AND METHODS

### Patients and Samples

From June 2014 to June 2016, 57 CRC tissues were collected. The diagnosis of CRC was confirmed by hematoxylin and eosin staining. Patient characteristics are shown in Supplementary Table 1. This study was approved by the Ethics Committee of the Affiliated Hospital of Qingdao University, and the investigations were carried out following the rules of the Declaration of Helsinki of 1975 (https://www.wma.net/policies-post/wma-declaration-of-helsinki-ethical-principles-for-medical-research-involving-human-subjects/), revised in 2013. Signed informed consent was obtained from all patients included in the study, and all the experiments were carried out in accordance with the National Health and Family Planning Commission of the PRC's guidelines.

### NGS-based assay

Tumor DNA was extracted from FFPE samples using a QIAamp DNA FFPE Tissue Kit and a Qiagen's DNEasy Blood and Tissue Extraction Kit (Qiagen), according to the manufacturer's instructions. Genomic DNA from 2ml peripheral blood was purified using a QIAamp DNA Blood Mini Kit (Qiagen). All FFPE tissue samples were reviewed by a qualified pathologist to ensure >70% tumor content. DNA purity and concentration were determined by the NanoDrop2000 spectrophotometer and Qubit 2.0 Fluorometer with Quant-IT dsDNA HS Assay Kit (Thermo Fisher Scientific), respectively. The quality of genomic DNA from tumor tissue and peripheral blood was assessed by agarose gel electrophoresis, and the size distribution of circulating DNA was evaluated on a 2100 Bioanalyzer using the DNA 1000 Kit (Agilent).

Library construction with tumor tissue DNA and paired peripheral blood DNA was performed using 1μg of DNA sheared by an ultrasonoscope to generate fragments with a peak of 250 bps, followed by end repair, A-tailing and ligation to the Illumina-indexed adapters according to the standard library construction protocol. Target enrichment was performed on a custom sequence capture-probe (Nimblegen, USA) that targeted 7,708 exons of 508 cancer-related genes and 78 introns from 19 genes recurrently rearranged in solid tumor, representing ∼1.7 Mb of the human genome in total (Supplementary Table 3). Sequencing was performed with 2×101 bp paired-end reads and 8-bp index reads on an Illumina Hiseq 2500 platform (Illumina, San Diego, USA) using the manufacturer's protocols.

Primary sequence data were first processed by filtering adaptor sequences and removing low-quality reads using the SOAPnuke software (http://soap.genomics.org.cn/) developed by BGI, and aligned to build hg19 of the NCBI reference genome assembly using BWA aligner v0.6.2-r126. PCR duplicate reads were removed by PICARD v1.98. Local realignment and base quality score recalibration were performed using GATK v2.3-9, and poorly mapped reads were removed based on the recalibration result. SNVs were detected by Mutect and SOMATK-SNV (developed by BGI, manuscript in preparation), and Indel (small insertions and deletions) were detected by GATK and SOMATK-INDEL (developed by BGI, manuscript in preparation). A minimal amplicon coverage of 300 was defined, and a variant allele frequency of 1% as theminimal threshold was used to provide reliable diagnostic analysis. CNV calling was done by CONTRA v2.0.4 [35]. We defined a gene as showing copy number gain when its coverage fold ratio was ≥2.0 and loss when ≤0.5.

### Statistical analysis

The experimental data are presented as the mean±SEM and were analyzed by a two-tailed Student's t test. The threshold of P < 0.05 was considered statistically significant.

## SUPPLEMENTARY MATERIALS TABLES






