# Supplementary material for: Targeted next generation sequencing in Chinese colorectal cancer patients guided anti-EGFR treatment and facilitated precision cancer medicine
Source: Oncotarget. 2017 Sep 27;8(62):105072–80. doi: 10.18632/oncotarget.21349 (PMC5739621; doi:10.18632/oncotarget.21349)
Supplement: Supplementary file 1 [file oncotarget-08-105072-s001.pdf]

Targeted next generation sequencing in Chinese colorectal cancer patients guided anti-EGFR treatment and facilitated precision cancer medicine

SUPPLEMENTARY MATERIALS

Supplementary Table 1: Clinical characteristics of the 57 colorectal cancer patients

Clinical characteristics (n=57)	
Age—years	
Median	60.4
Range	35-83
Sex—no.	
Male	32(56.1%)
Female	25(43.9%)
Tissue sample source	
Primary	49(90.9%)
Liver metastases	3(9.1%)
Lung metastases	3(9.1%)
Ovary metastases	2(9.1%)
Stage	
II	5(8.8%)
III	9(15.8%)
IV	43(75.4%)

Supplementary Table 2: Sequencing data of the CRC patients by NGS

See Supplementary File 1

Supplementary Table 3: Gene list of 508 genes panel detected by NGS assay in this study

See Supplementary File 2
