# Supplementary material for: Targeted next generation sequencing in Chinese colorectal cancer patients guided anti-EGFR treatment and facilitated precision cancer medicine
Source: Oncotarget. 2017 Sep 27;8(62):105072–80. doi: 10.18632/oncotarget.21349 (PMC5739621; doi:10.18632/oncotarget.21349)
Supplement: Supplementary file 3 [file oncotarget-08-105072-s003.doc]

**Supplementary Table S3.** Gene list of 508 genes panel detected by NGS assay in this study

| ABL1 | C1R | DIS3 | FGF19 | HSPA4 | MIR142 | PAX5 | RB1 | SRSF2 |
| --- | --- | --- | --- | --- | --- | --- | --- | --- |
| ABL2 | C1S | DNMT1 | FGF23 | IDH1 | MITF | PBRM1 | REL | SSTR2 |
| ACVR1B | CARD11 | DNMT3A | FGF3 | IDH2 | MLH1 | PCBP1 | RET | STAG2 |
| ACVR2A | CASP8 | DOT1L | FGF4 | IFNAR1 | MLH3 | PCM1 | RHEB | STAT4 |
| AJUBA | CBFB | DUSP6 | FGF6 | IFNAR2 | MLL | PDGFRA | RICTOR | STAT5B |
| AKT1 | CBL | EDNRA | FGF7 | IGF1 | MLL2 | PDGFRB | RNASEL | STK11 |
| AKT2 | CBLB | EGFR | FGFR1 | IGF1R | MLL3 | PDK1 | RNF43 | SUFU |
| AKT3 | CBR1 | EGR3 | FGFR2 | IGF2 | MLL4 | PHF6 | ROBO1 | SUZ12 |
| ALK | CCND1 | EIF4A2 | FGFR3 | IKBKB | MPL | PIGF | ROBO2 | SYK |
| ALOX12B | CCND2 | ELAC2 | FGFR4 | IKBKE | MRE11A | PIK3C2A | ROS1 | TAF1 |
| ANGPT1 | CCND3 | ELF3 | FH | IKZF1 | MS4A1 | PIK3C2B | RPA1 | TBL1XR1 |
| ANGPT2 | CCNE1 | EML4 | FLCN | IL7R | MSH2 | PIK3C2G | RPL22 | TBX3 |
| APC | CD79A | EP300 | FLT1 | INHBA | MSH3 | PIK3C3 | RPL5 | TEK |
| APCDD1 | CD79B | EPCAM | FLT3 | IRF4 | MSH4 | PIK3CA | RPS14 | TERT |
| AR | CDC25C | EPHA2 | FLT4 | IRS2 | MSH5 | PIK3CB | RPS6KB1 | TET2 |
| ARAF | CDC42 | EPHA3 | FNTA | ITGB2 | MSH6 | PIK3CG | RPTOR | TFG |
| ARFRP1 | CDC73 | EPHA5 | FOXA1 | JAK1 | MSR1 | PIK3R1 | RUNX1 | TGFBR2 |
| ARHGAP35 | CDH1 | EPHB1 | FOXA2 | JAK2 | MTOR | PIK3R2 | RUNX1T1 | TIPARP |
| ARID1A | CDK12 | EPHB2 | FOXL2 | JAK3 | MUC1 | PLK1 | RXRA | TLR4 |
| ARID1B | CDK2 | EPHB6 | FPGS | JUN | MUTYH | PML | RXRB | TMEM127 |
| ARID2 | CDK4 | EPPK1 | FUBP1 | KAT6A | MYC | PMS1 | RXRG | TNFAIP3 |
| ARID5B | CDK6 | ERBB2 | FYN | KDM5A | MYCL1 | PMS2 | SDHAF2 | TNFRSF14 |
| ASXL1 | CDK8 | ERBB3 | GAB2 | KDM5C | MYCN | PNRC1 | SDHB | TNFRSF8 |
| ATM | CDKN1A | ERBB4 | GATA1 | KDM6A | MYD88 | POLQ | SDHC | TNFSF11 |
| ATR | CDKN1B | ERCC2 | GATA2 | KDR | NAV3 | PPP2R1A | SDHD | TNFSF13B |
| ATRX | CDKN2A | ERCC3 | GATA3 | KEAP1 | NBN | PRDM1 | SEMA3A | TOP1 |
| AURKA | CDKN2B | ERG | GID4 | KIF1B | NCOA1 | PRKAA1 | SEMA3E | TOP2A |
| AURKB | CDKN2C | ESR1 | GNA11 | KIF5B | NCOA2 | PRKAR1A | SETBP1 | TOP2B |
| AXIN1 | CDX2 | ETV1 | GNA13 | KIT | NCOR1 | PRKCA | SETD2 | TP53 |
| AXIN2 | CEBPA | ETV6 | GNAQ | KLF4 | NEK11 | PRKCB | SF1 | TRAF7 |
| AXL | CFLAR | EWSR1 | GNAS | KLHL6 | NF1 | PRKCG | SF3B1 | TSC1 |
| B2M | CHD1 | EXT1 | GNRHR | KRAS | NF2 | PRKDC | SH2B3 | TSC2 |
| B4GALT3 | CHD2 | EXT2 | GPR124 | LCK | NFE2L2 | PRSS8 | SIN3A | TSHR |
| BACH1 | CHD4 | EZH2 | GRIN2A | LIMK1 | NFE2L3 | PSMB1 | SLAMF7 | TSHZ2 |
| BAK1 | CHEK1 | FAM123B | GRM3 | LRRK2 | NFKBIA | PSMB2 | SLC4A1 | TSHZ3 |
| BAP1 | CHEK2 | FAM46C | GSK3B | LYN | NKX2-1 | PSMB5 | SLIT2 | TUBA1A |
| BARD1 | CHUK | FANCA | H3F3A | MALAT1 | NKX3-1 | PTCH1 | SMAD2 | TUBB |
| BCL2 | CIC | FANCC | H3F3C | MAP2K1 | NOTCH1 | PTCH2 | SMAD3 | TUBD1 |
| BCL2A1 | CRBN | FANCD2 | HCK | MAP2K2 | NOTCH2 | PTEN | SMAD4 | TUBE1 |
| BCL2L1 | CREBBP | FANCE | HDAC1 | MAP2K4 | NOTCH3 | PTP4A3 | SMARCA1 | TUBG1 |
| BCL2L11 | CRIPAK | FANCF | HDAC2 | MAP3K1 | NOTCH4 | PTPN11 | SMARCA4 | TYR |
| BCL2L2 | CRKL | FANCG | HDAC3 | MAP3K13 | NPM1 | PTPRD | SMARCB1 | U2AF1 |
| BCL6 | CRLF2 | FANCI | HDAC4 | MAPK1 | NR3C1 | RAC1 | SMARCD1 | USP9X |
| BCOR | CROT | FANCL | HDAC6 | MAPK3 | NRAS | RAC2 | SMC1A | VEGFA |
| BCORL1 | CSF1R | FANCM | HDAC8 | MAPK8 | NSD1 | RAD21 | SMC3 | VEGFB |
| BCR | CTCF | FAT3 | HGF | MAPK8IP1 | NTRK1 | RAD50 | SMO | VEZF1 |
| BLM | CTLA4 | FBXW7 | HIF1A | MAX | NTRK2 | RAD51 | SOCS1 | VHL |
| BMPR1A | CTNNA1 | FCGR1A | HIST1H1C | MC1R | NTRK3 | RAD51B | SOX10 | WHSC1L1 |
| BRAF | CTNNB1 | FCGR2A | HIST1H2BD | MCL1 | NUP93 | RAD51C | SOX17 | WISP3 |
| BRCA1 | CUL4A | FCGR2B | HIST1H3B | MDM2 | PAK3 | RAD51D | SOX2 | WWP1 |
| BRCA2 | CUL4B | FCGR2C | HNF1A | MDM4 | PAK7 | RAD52 | SOX9 | XIAP |
| BRIP1 | CYLD | FCGR3A | HRAS | MECOM | PALB2 | RAD54L | SPEN | XPA |
| BTG1 | CYP17A1 | FCGR3B | HRH2 | MED12 | PARP1 | RAF1 | SPOP | XPC |
| BTK | DAXX | FGF10 | HSD17B3 | MEF2B | PARP2 | RARA | SPRY4 | XPO1 |
| C11orf30 | DDR1 | FGF12 | HSD3B2 | MEN1 | PARP3 | RARB | SRC | XRCC3 |
| C1QA | DDR2 | FGF14 | HSP90AA1 | MET | PARP4 | RARG | SRD5A2 | YES1 |
| ZNF217 | ZNF703 | ZRSR2 | WT1 |  |  |  |  |  |
